# Metabolomic Analysis of Vitamin E Supplement Use in the Prostate, Lung, Colorectal, and Ovarian Cancer Screening Trial

**DOI:** 10.3390/nu15132836

**Published:** 2023-06-22

**Authors:** Jungeun Lim, Hyokyoung G. Hong, Stephanie J. Weinstein, Mary C. Playdon, Amanda J. Cross, Rachael Stolzenberg-Solomon, Neal D. Freedman, Jiaqi Huang, Demetrius Albanes

**Affiliations:** 1 Division of Cancer Epidemiology and Genetics, National Cancer Institute, National Institutes of Health, Bethesda, MD 20892, USA; jungeun.lim@nih.gov (J.L.); grace.hong@nih.gov (H.G.H.); weinstes@mail.nih.gov (S.J.W.); rachael.solomon@nih.gov (R.S.-S.); freedmanne@mail.nih.gov (N.D.F.); jiaqi.huang@live.com (J.H.); 2 University of Utah and Cancer Control and Population Sciences Program, Department of Nutrition and Integrative Physiology, Huntsman Cancer Institute, University of Utah, Salt Lake City, UT 84112, USA; mary.playdon@hci.utah.edu; 3 Department of Epidemiology and Biostatistics, School of Public Health, Imperial College London, London SW7 2AZ, UK; amanda.cross@imperial.ac.uk; 4 Cancer Screening & Prevention Research Group, Department of Surgery & Cancer, Imperial College London, London SW7 2AZ, UK

**Keywords:** vitamin E supplementation, vitamin E dosage, serum metabolomics, prostate cancer

## Abstract

The effects of vitamin E supplementation on cancer and other chronic diseases are not clear. We compared the serum metabolomic profile of differing vitamin E dosages in order to re-examine the previously observed changes in a novel C_22_ lactone sulfate compound, androgenic steroids, and other metabolites. A total of 3409 women and men previously selected for metabolomics studies in the PLCO Cancer Screening Trial were included in this investigation. Serum metabolites were profiled using ultrahigh-performance liquid and gas chromatography/tandem mass spectrometry. Seventy known metabolites including C_22_ lactone sulfate and androgens were significantly associated with vitamin E supplementation. In the sex-stratified analysis, 10 cofactors and vitamins (e.g., alpha-CEHC sulfate and alpha-CEHC glucuronide), two carbohydrates (glyceric and oxalic acids), and one lipid (glycocholenate sulfate) were significantly associated with vitamin E dose in both males and females (FDR-adjusted *p*-value < 0.01). However, the inverse association between C_22_ lactone sulfate and daily vitamin E supplementation was evident in females only, as were two androgenic steroids, 5-androstenediol and androsterone glucuronide. Our study provides evidence of distinct steroid hormone pathway responses based on vitamin E dosages. Further studies are needed to gain biological insights into vitamin E biochemical effects relevant to cancer and other chronic diseases.

## 1. Introduction

Alpha-tocopherol is a common dietary and supplemental vitamin E compound that is preferentially taken up by the liver and distributed throughout the body, serving as a major lipid antioxidant [1]. Epidemiological studies and laboratory experiments indicate inverse associations between vitamin E intake from dietary sources and/or supplements and cancer risk [2,3]. Along with increased interest in research on cancer treatment, it has also been reported that vitamin E is involved in the amelioration of side-effects from chemotherapy and radiation therapy [4,5]. Alpha-tocopherol may prevent cancer by inhibiting cell proliferation and angiogenesis, inducing apoptosis and enhancing immune function [6,7,8]. In a randomized controlled trial (RCT), a 50 IU daily dose of vitamin E (as alpha-tocopheryl acetate) resulted in significant 32% and 40% reductions in prostate cancer incidence and mortality, respectively [9]. However, subsequent RCTs using higher vitamin E dosages of 200 IU or 400 IU daily showed contradictory (17% increased) or insignificant effects on prostate cancer incidence [10,11].

To gain biological insights into vitamin E biochemical effects relevant to cancer, including the divergent prostate cancer findings in those RCTs, a metabolomic analysis was conducted in another RCT of high-dose vitamin E, which showed the expected increases in serum vitamin E-related metabolites including alpha-carboxyethyl hydrochroman (CEHC) sulfate and alpha-tocopherol in response to the 400 IU/day vitamin E, as well as significant reductions in a novel C_22_ lactone sulfate and androgen metabolites [12]. A strong correlation was also observed between the changes in androgenic steroid metabolites and the vitamin E supplement-associated reduction in C_22_ lactone sulfate in the 400 IU/day RCT only. As for the other cancer sites, in vitro studies and animal models of breast cancer supplemented with tocopherol or tocotrienol vitamers showed antitumor and chemo-preventive activity [13], and inverse associations have been observed between vitamin E consumption and risk of bladder and esophageal cancer [14,15].

In the present cross-sectional investigation, we compared the serum metabolomic profile of low and high-dose vitamin E supplementation in order to re-examine the previously observed changes in a novel C_22_ lactone sulfate compound, androgenic steroids, and other metabolites.

## 2. Materials and Methods

### 2.1. Study Population

Data for this study were derived from an RCT, the Prostate, Lung, Colorectal, and Ovarian (PLCO) Cancer Screening Trial. The design of this trial was described previously [16]. In brief, participants aged 55 to 74 years were recruited from 10 study centers across the US between September 1993 and June 2001. Participants provided written informed consent, and the study was approved by the institutional review boards of the US National Cancer Institute and the ten PLCO screening centers.

**Figure 1:**
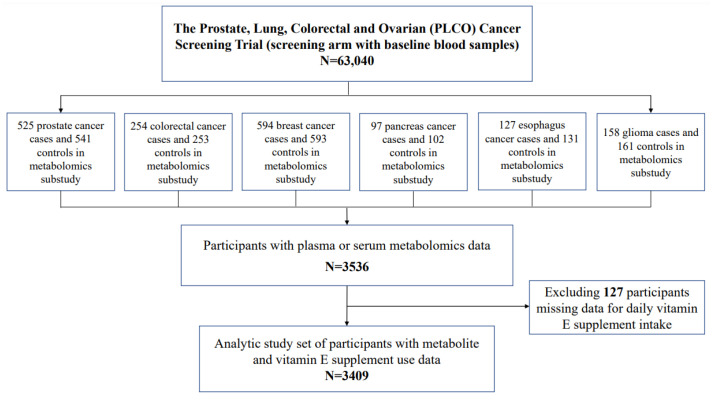
Participant flowchart.

The present investigation includes participants in the PLCO screening arm who were previously selected for seven metabolomic nested case–control studies of cancer risk. These included two studies of prostate (525 cases and 541 controls) [17] (and unpublished), and one study each of colorectum (254 cases and 253 controls) [18], breast (594 cases and 593 controls) [19], pancreas (97 cases and 102 controls) [20], esophagus (127 cases and 131 controls; unpublished), and glioma (158 cases and 161 controls; unpublished). After excluding 127 participants missing data for daily vitamin E supplement intake, a total of 3409 participants including 1837 males and 1572 females with serum metabolomic data were included in the present analysis (Figure 1). All vitamin supplement and metabolomic data were from baseline (i.e., before cancers were diagnosed).

### 2.2. Serum and DATA Collection

At enrollment, participants completed questionnaires regarding behavioral and lifestyle information, including use of supplemental vitamin E and multivitamins, and smoking status (i.e., never, current, and former), and blood (of variable fasting states) was collected and processed to serum. Daily vitamin E supplement dosages were calculated from single-vitamin and multivitamin supplementation questions. Regarding the use of vitamin E capsules/tablets, people responded to the question ‘what dose per day did/do you usually take? (i.e., 100 IU, 200 IU, 400 IU, 800 IU, 1000 + IU, do not know)’. Participants were asked to answer the following question for multivitamin use: How many pills did/do you usually take? (i.e., <2/week, 2–4/week, 5–6/week, 1/day, and 2+/day). Dosage values for multivitamins were derived from the Third National Health and Nutrition Examination Survey (NHANES III) database (e.g., 30 IU).

### 2.3. Metabolomic Analysis

Serum metabolites were assayed at Metabolon, Inc. (Durham, NC, USA) using a high-resolution, accurate mass platform of ultrahigh-performance liquid chromatography/mass spectrometry and gas chromatograph/mass spectrometry (GC–MS). The details of the analytical platform that integrated the chemical analysis were described in [21,22,23]. A total of 2356 metabolites were detected. After excluding those with >10% missing values, 1713 compounds (including 1155 known metabolites) remained for analysis. On the basis of the existing literature, metabolites were categorized across eight mutually exclusive chemical classes: amino acids, carbohydrates, cofactors and vitamins, energy metabolites, lipids, nucleotides, peptides, and xenobiotics [24].

### 2.4. Statistical Analysis

According to the daily vitamin E supplementation dosages and multivitamin use, we created three daily dosage categories: 0 IU, 4.3–<400 IU, and ≥400 IU. ANCOVA analysis was used to identify metabolites showing significant differences among daily vitamin E supplementation levels, adjusting for potential confounders such as age (continuous), sex (male or female), body weight (continuous), smoking status (never, current, and former), and multivitamin use (yes or no). Including a variable for case status (case, control) in the model did not materially alter the vitamin E association with any metabolite (i.e., <10% change).

Metabolites associated with daily supplemental vitamin E intake were determined for all participants combined and separately for males and females. Sensitivity analyses were conducted to additionally adjust for alcohol and dietary consumption (i.e., whole grains, fruits, eggs, dairy products, fish, and meats). Considering the higher vitamin E dose of therapeutic multivitamins (e.g., 60 IU), sensitivity analyses were also conducted that excluded therapeutic multivitamin use. Models were also stratified by age (63 years), body mass index (BMI, 27.2 kg/m^2^ for males and 26.6 kg/m^2^ for women), body weight (86.2 kg for males and 70.3 kg for females) based on median values, and smoking status (never, current, and former).

To account for multiple comparisons, the false discovery rate (FDR) using the Benjamini and Hochberg approach [25] was used to help to avoid type I errors [26]. All analyses were performed using SAS statistical software version 9.4 (SAS Institute, Cary, NC, USA). All reported *p*-values were two-sided. The null hypothesis of no difference was rejected if *p*-values were <0.05.

## 3. Results

Baseline characteristics of the study participants according to daily supplemental vitamin E dosage are presented in Table 1. The study population included 1837 (53.9%) males and 1572 (46.1%) females. Of these, 835 males and 919 females reported taking a single vitamin E supplement or multivitamin, with the median supplemental vitamin E intake being 200 IU and 400 IU, respectively.

Among the 1713 compounds measured, 70 known metabolites, including alpha-tocopherol and its metabolites, C_22_ lactone sulfate, and androgen metabolites, were significantly associated with daily supplemental vitamin E intake and showed some dose–response gradients (Table 2). Metabolites in tocopherol metabolism such as alpha-CEHC, alpha-CEHC-glucuronide, and alpha-CEHC sulfate were significantly positively associated with supplemental vitamin E after multiple comparisons correction (FDR-adjusted *p*-value <0.001 for each metabolite). C_22_ lactone sulfate was significantly lower with increasing vitamin E dosage (betas of −0.04 and −0.17 and *p*-values 0.500 and <0.001 for the <400 and >400 IU categories, respectively) (Table 2). Results were consistent when we additionally adjusted for dietary and alcohol consumption. Nine sex steroids, including seven androgenic steroids, were inversely and significantly associated with vitamin E supplement use (FDR-adjusted *p*-value <0.01 for each metabolite; Table 2).

In the sex-stratified analysis, 13 metabolites including 10 cofactors and vitamins such as alpha-CEHC sulfate and alpha-CEHC glucuronide, two carbohydrates (glyceric acid and oxalic acid), and glycocholenate sulfate were significantly related to vitamin E supplementation in both males and females (FDR-adjusted *p*-value < 0.01) (Table 3). An inverse association between C_22_ lactone sulfate and dose of supplemental vitamin E was apparent only in females (FDR-adjusted *p*-value = 0.046, *p* for interaction = 0.0218). This finding was unchanged when we additionally adjusted for dietary and alcohol consumption. 5-Androstenediol and androsterone glucuronide were also inversely and significantly associated with vitamin E intake only in females (*p* for interaction = 0.0214 and 0.0008, respectively). In the body weight-stratified analysis, C_22_ lactone sulfate (FDR-adjusted *p*-value = 0.139) and two androgen metabolites, androsterone glucuronide (FDR-adjusted *p*-value = 0.006) and 5-androstenediol (FDR-adjusted *p*-value = 0.064), were significantly related to vitamin E supplementation only in females >70.3 kg (the median) (data not shown). There were no apparent interactions of the vitamin E-metabolite associations with age, BMI, or smoking status. Our findings were not materially altered when we excluded therapeutic multivitamin users.

## 4. Discussion

In this first metabolomic analysis of vitamin E supplementation in both males and females, along with the expected increases in alpha-tocopherol and its metabolites, a novel C_22_ lactone sulfate compound and androgenic steroid metabolites were significantly lower in vitamin E supplement users, with some indication of a dose–response relationship. Interestingly, the findings were stronger in females than in males, whereas a previous RCT-based study showed a strong signal for C_22_ lactone sulfate and androgens in males [10].

Previous metabolomic studies of different cancers having a focus on vitamin E are limited. Regarding the biological insights into vitamin E biochemical effects (including sex steroids) relevant to cancers, the delta-tocopherol vitamers significant in our study showed antitumor and adjuvant chemopreventive potential in breast cancer cells [13]. Although prospective studies in healthy women have consistently shown a strong relationship between high serum androgen levels and increased risk of developing ER-positive breast cancers [27,28], the link between androgen receptor (AR) positivity and improved outcomes in ER-positive tumors has revealed the beneficial effect of androgens [29]. The inhibition of glioma cell proliferation induced by tocopherols was also observed in experimental studies [30], but inconsistent associations were found for colon cancer risk and vitamin E supplements [31] even though a meta-analysis of case–control studies showed lower concentrations of serum vitamin E in patients with colorectal cancer compared with healthy controls [32]. Regarding esophageal cancer risk, an in vivo study suggested vitamin E may suppress *N*-nitrosomethylbenzylamine-induced carcinogenesis in the rat esophagus by blocking activation of nuclear factor-kappa B (NF-κB) and abnormal arachidonic acid metabolism [33]. Little is known regarding vitamin E-related metabolites and pancreatic cancer risk.

With regard to prostate cancer risk, an early large prevention trial of male Finnish smokers showed significant 32% and 40% reductions in prostate cancer incidence and mortality, respectively, in response to a modest daily dose of 50 IU dl-alpha-tocopheryl acetate. Subsequent ‘confirmator’” trials using substantially higher vitamin E dosages of 200 and 400 IU/day yielded contradictory outcomes (e.g., 17% increased incidence) [11] and no effects on prostate cancer [10]. Although the present study provided little evidence for distinct steroid hormone pathway changes based on varying vitamin E dosages in males which could have direct relevance to prostate cancer risk, changes in androgenic steroid metabolites were strongly correlated with the high-dose vitamin E supplement-associated change in C_22_ lactone sulfate in the Vitamin E Atherosclerosis Prevention Study (VEAPS) trial [12], with similar, albeit more modest, associations in females in the present study. Further investigation is needed to more fully understand the interrelationships among C_22_ lactone sulfate, androgenic hormones, and vitamin E supplementation, including possible sex differences.

The molecular structure of C_22_ lactone sulfate suggests possible involvement in the lanosterol synthase pathway [10]. One genome-wide association study (GWAS) showed that C_22_ lactone sulfate variation was associated with cytochrome P450 (CYP) 3A5 on chromosome 7 which encodes a member of the CYP superfamily of enzymes involved in drug metabolism (including ibuprofen) and synthesis of cholesterol, steroids, and other lipids related to the lipid-lowering efficacy of simvastatin [34,35,36]. Metabolism of vitamin E involves CYP enzymes, with CYP3A4 and CYP4F2 suggested to be involved in tocopherol degradation and drug metabolism, the latter being substrate-induced through activation of the pregnane X receptor (PXR) [37]. Tocopherols and tocotrienols induce the expression of a PXR-driven reporter gene and endogenous CYP3A4 and CYP3A5. With respect to prostate cancer, higher PXR expression in cancerous versus normal tissues has been observed, and PXR activation is associated with increased tumor progression and resistance to the chemotherapeutic drugs [38,39,40]. C_22_ lactone sulfate has also been associated with genetic variation of the protein-coding SLCO1B1 which is involved in hepatic uptake of statins [41]. Since prospective and registry-based studies support a lower risk of advanced and fatal prostate cancer in statin users relative to nonusers, as well as better outcomes among prostate cancer patients, C_22_ lactone sulfate may be related to the statin–prostate cancer association [42]. Functional studies of this compound are needed to elucidate the precise biochemical actions and pathways involved.

It is interesting to note that significant inverse associations between androgenic steroids and vitamin E intake were observed only in females in our study. The results were unchanged after additional adjustment for use of hormone replacement therapy. Experiments have shown that alpha-tocopheryl succinate can suppress androgen receptor (AR) expression by means of transcriptional and posttranscriptional modulation [43]. Results from NHANES III showed an inverse association between serum alpha-tocopherol and circulating testosterone, estradiol, and sex hormone-binding globulin, but only in males who smoked [44]. The effect of sex on the relationship between androgenic steroid metabolites and vitamin E should be re-examined within other populations.

This is the first metabolomic analysis of vitamin E supplementation in males and females. We utilized an untargeted metabolomic platform exhibiting high laboratory validity and reproducibility. More than 1700 metabolites reflecting a broad array of biochemicals and biological pathways were identified. Application of a rigorous FDR correction for multiple comparisons revealed several individual metabolites related to higher vitamin E dosage, including C_22_ lactone sulfate and androgens. Although use of self-reported supplementation data afforded us a less stringent test of the vitamin E/metabolite hypothesis as compared with the previous controlled trial analysis in males [12], dose–response associations between alpha-tocopherol and its metabolites and increasing vitamin E dosages support the validity of our exposure measure; that is, elevated alpha-CEHC and alpha-tocopherol, and decreased delta-tocopherol reflected the increase in serum vitamin E from higher supplement dosage. Including participants who had been previously selected for nested case–control studies of metabolomics in the PLCO trial is a potential limitation; however, supplement use data and metabolite measurements were based on baseline reports and serum up to two decades prior to cancer diagnoses. Furthermore, our primary findings were similar when we restricted the analysis to the control participants. Despite the large number of measured compounds, other unmeasured metabolites or biochemical pathways related to high-dose vitamin E supplementation may not have been identified using the assay platform utilized. It is also possible that some findings of xenobiotic drugs (e.g., 2-methoxyacetaminophen glucuronide) were confounded by other drug intake such as acetaminophen, and other diet biomarkers may have affected the findings although we adjusted for potential confounding factors.

In conclusion, vitamin E supplementation was associated with significantly lower serum C_22_ lactone sulfate and androgenic steroid metabolites in PLCO. The associations were evident primarily in females, however. Although our study provides some evidence of distinct steroid hormone pathway responses based on vitamin E dosages that could have direct relevance to previous trial findings for prostate cancer, they require re-examination in other populations of males and females, including especially in vitamin E clinical trial settings, where possible. Since experimental evidence does support inhibitory effects of lactone-containing metabolites and lactone-based derivatives on cancer cell line growth [45,46,47], re-examination in other populations along with further elucidation of the interrelationships and biochemical pathways among C_22_ lactone sulfate and vitamin E would be helpful to gain greater biological insights into the biochemical effects of vitamin E relevant to cancer.

## Figures and Tables

**Table 1:** Participant baseline characteristics by daily supplemental vitamin E intake in the PLCO Study ^1^.

	All Participants			Men			Women		
	0 IU	4.3–<400 IU IU	≥400 IU	0 IU	4.3–<400 IU IU	≥400 IU	0 IU	4.3–<400 IU IU	≥400 IU
*n*	1655	872	882	1002	446	389	653	426	493
Age, years	63.4 ± 5.0	63.1 ± 5.3	63.0 ± 5.2	63.6 ± 5.0	62.8 ± 5.3	63.0 ± 5.1	63.3 ± 5.1	63.3 ± 5.4	62.9 ± 5.2
Race, %									
White, non-Hispanic	79.8	86.6	85.7	72.8	81.6	77.6	90.5	91.8	92.1
Black, non-Hispanic	16.7	8.9	9.1	24.2	13.5	17.0	5.2	4.2	2.8
Other races combined	3.6	4.5	5.2	3.1	4.9	5.4	4.3	4.0	5.1
Height, cm	172.1 ± 9.9	171.0 ± 10.1	169.9 ± 9.8	177.9 ± 7.1	178.2 ± 7.0	178.1 ± 7.0	163.2 ± 6.3	163.5 ± 6.9	163.5 ± 6.4
Weight, kg	82.9 ± 16.7	79.9 ± 15.5	78.5 ± 16.5	88.7 ± 15.2	86.6 ± 14.0	88.0 ± 14.8	74.0 ± 15.0	72.8 ± 13.8	71.0 ± 13.7
Body mass index, kg/m^2^	27.9 ± 4.8	27.3 ± 4.6	27.1 ± 4.7	28.0 ± 4.5	27.3 ± 4.0	27.7 ± 4.1	27.8 ± 5.3	27.3 ± 5.2	26.6 ± 5.1
Tobacco smoking status, %									
Never smoker	44.5	47.7	47.4	35.7	36.8	35.5	57.9	59.2	56.8
Former smoker	9.7	6.2	7.4	10.9	6.5	8.7	7.8	5.9	6.3
Current smoker	45.9	46.1	45.2	53.4	56.7	55.8	34.3	35.0	36.9
Multivitamin use, %	0	95.8	74.3	0	95.7	72.8	0	95.8	75.5

Abbreviations: PLCO = Prostate, Lung, Colorectal, and Ovarian Cancer Screening Trial. ^1^ Mean ± standard deviation (all values except as noted).

**Table 2:** Metabolites associated with daily supplemental vitamin E intake in the PLCO Study (FDR-adjusted *p*-value <0.1) ^1,2^.

					4.3–<400 IU IU		≥400 IU	
Metabolite	Chemical Class	Sub-Pathway	F-Value	FDR- Adjusted *p*-Value	Effect Size (β)	*p*-Value	Effect Size (β)	*p*-Value
Alpha-CEHC	Cofactors and vitamins	Tocopherol metabolism	364.47	<0.001	0.62	<0.001	1.80	<0.001
Alpha-CEHC-glucuronide	Cofactors and vitamins	Tocopherol metabolism	338.54	<0.001	0.66	<0.001	1.90	<0.001
Alpha-Tocopherol	Cofactors and vitamins	Tocopherol metabolism	318.33	<0.001	0.18	<0.001	0.44	<0.001
Alpha-CEHC sulfate	Cofactors and vitamins	Tocopherol metabolism	214.10	<0.001	1.09	<0.001	2.38	<0.001
Pantothenic acid	Cofactors and vitamins	Pantothenate and CoA metabolism	53.15	<0.001	0.17	<0.001	0.29	<0.001
L-Threonic acid	Cofactors and vitamins	Ascorbate and aldarate metabolism	43.46	<0.001	0.11	0.002	0.27	<0.001
4-Pyridoxic acid	Cofactors and vitamins	Vitamin B6 metabolism	43.16	<0.001	0.14	0.021	0.43	<0.001
Delta-Tocopherol	Cofactors and vitamins	Tocopherol metabolism	34.05	<0.001	−0.20	0.008	−0.49	<0.001
Glyceric acid	Carbohydrate	Glycolysis, gluconeogenesis, pyruvate metabolism	30.86	<0.001	0.08	<0.001	0.13	<0.001
Oxalic acid	Carbohydrate	Glyoxylate and dicarboxylate metabolism	28.66	<0.001	0.10	0.013	0.24	<0.001
Pyridoxal	Cofactors and vitamins	Vitamin B6 metabolism	22.79	<0.001	0.03	0.79	0.45	<0.001
Hydroxypropanedioic acid	Xenobiotics	Food component/plant	16.12	<0.001	0.12	0.002	0.18	<0.001
N1-Methyl-2-pyridone-5-carboxamide	Cofactors and vitamins	Nicotinate and nicotinamide metabolism	15.21	<0.001	0.08	0.041	0.18	<0.001
Glycocholenate sulfate	Lipid	Bile acid metabolism	15.16	<0.001	−0.10	0.001	−0.15	<0.001
Beta-Tocopherol	Cofactors and vitamins	Tocopherol metabolism	14.46	<0.001	−0.26	0.020	−0.49	<0.001
Hydroxy-carboxy-4-methyl-5-propyl-2-furanpropionic acid (CMPF)	Lipid	Fatty acid, dicarboxylate	14.36	<0.001	−0.12	0.043	0.13	0.013
N1-Methyl-4-pyridone-3-carboxamide	Cofactors and vitamins	Nicotinate and nicotinamide metabolism	11.37	0.001	0.13	0.004	0.18	<0.001
Isovaleric acid	Lipid	Fatty acid metabolism	10.98	0.001	0.00	0.882	0.09	<0.001
4-Androsten-3alpha,17alpha-diol monosulfate (2) or androstenediol (3alpha, 17alpha) monsulfate (2)	Lipid	Androgenic steroids	10.34	0.002	−0.15	0.065	−0.18	0.012
C_22_ lactone sulfate (X_12063)	Partially characterized	Partially characterized	8.99	0.007	−0.04	0.500	−0.17	<0.001
Decanoylcarnitine	Lipid	Carnitine metabolism	8.28	0.013	0.01	0.885	−0.11	0.003
Docosahexaenoic acid	Lipid	Essential fatty acid	8.19	0.014	−0.04	0.228	0.06	0.031
Androsterone glucuronide	Lipid	Androgenic steroids	8.08	0.016	−0.19	0.008	−0.26	<0.001
Phosphate	Energy	Oxidative phosphorylation	8.02	0.016	0.06	<0.001	0.06	<0.001
L-Octanoylcarnitine	Lipid	Carnitine metabolism	7.86	0.017	0.01	0.806	−0.10	0.004
*N*-Stearoyltaurine or *N*-Stearoyl taurine	Lipid	Endocannabinoid	7.86	0.018	0.07	0.244	0.01	0.863
Sphingomyelin (d17:1/14:0, d16:1/15:0)	Lipid	Sphingomyelins	7.67	0.020	−0.15	0.014	−0.10	0.063
Eicosapentaenoic acid	Lipid	Essential fatty acid	7.55	0.021	0.01	0.733	0.10	0.002
5alpha-Androstan-3alpha,17alpha-diol disulfate	Lipid	Sterol/steroid	7.50	0.021	0.03	0.733	−0.05	0.492
S-Allylcysteine	Xenobiotics	Food component/plant	7.29	0.026	−0.19	0.111	0.17	0.100
3-Carboxy-4-methyl-5-propyl-2-furanpropionic acid (CMPF)	Lipid	Fatty acid, dicarboxylate	7.25	0.026	−0.08	0.210	0.11	0.054
L-Aspartic acid	Amino acid	Alanine and aspartate metabolism	7.19	0.027	0.01	0.663	−0.05	0.010
Monoglyceride (18:1(9Z)/0:0/0:0)	Lipid	Monoacylglycerol	7.18	0.027	0.03	0.594	0.01	0.760
L-Urobilin	Cofactors and vitamins	Hemoglobin and porphyrin metabolism	7.05	0.030	−0.02	0.799	−0.24	0.003
L-Glutamine	Amino acid	Glutamate metabolism	7.02	0.030	−0.02	0.032	−0.03	<0.001
Sphingomyelin (d17:1/16:0, d18:1/15:0, d16:1/17:0) or sphingomyelin (d18:1/15:0, d16:1/17:0)	Lipid	Sphingomyelins	6.90	0.034	−0.08	0.001	−0.09	<0.001
Tauro-b-muricholic acid	Lipid	Primary bile acid metabolism	6.82	0.034	0.18	0.064	0.30	<0.001
Lactosylceramide (d18:1/22:0)	Lipid	Lactosylceramides (LCER)	6.87	0.034	−0.19	<0.001	−0.11	0.008
2-Methoxyacetaminophen glucuronide	Xenobiotics	Drug	6.80	0.034	0.42	0.004	0.22	0.090
9-Hexadecenoylcarnitine	Lipid	Fatty acid metabolism (acyl carnitine, monounsaturated	6.75	0.036	0.04	0.393	0.02	0.585
Sphingomyelin (d17:2/16:0, d18:2/15:0)	Lipid	Sphingomyelins	6.78	0.036	−0.17	0.004	−0.12	0.013
Phosphatidylcholine (P-16:0/18:2)	Lipid	Plasmalogen	6.67	0.038	−0.11	0.001	−0.09	0.002
Homo-L-Arginine	Amino acid	Urea cycle; arginine and proline metabolism	6.54	0.042	−0.11	0.105	0.08	0.147
Pregnanediol	Lipid	Sterol/steroid	6.47	0.044	−0.07	0.126	−0.14	0.001
5alpha-Androstan-3alpha,17beta-diol disulfate	Lipid	Sterol/steroid	6.46	0.044	−0.11	0.192	−0.18	0.013
(R)C(S)S-Alliin	Xenobiotics	Food component/plant	6.27	0.051	−0.17	0.177	0.19	0.096
Oxypurinol	Xenobiotics	Drug—metabolic	6.14	0.057	−0.66	0.002	−0.16	0.371
L-Serine	Amino acid	Glycine, serine and threonine metabolism	6.13	0.057	−0.04	0.010	−0.04	0.001
O-Desmethyltramadol	Xenobiotics	Drug—analgesics, anesthetics	6.04	0.063	−0.13	0.008	−0.07	0.137
Dimethyl sulfone	Xenobiotics	Chemical	5.89	0.070	0.06	0.426	0.20	0.002
O-Phosphoethanolamine	Lipid	Phospholipid metabolism	5.88	0.070	−0.10	0.239	0.13	0.090
cis-4-Decenoate	Lipid	Medium-chain fatty acid	5.87	0.070	−0.03	0.668	−0.11	0.069
L-Methionine	Amino acid	Cysteine, methionine, SAM, taurine metabolism	5.86	0.070	0.01	0.662	0.00	0.807
3b,17a-Dihydroxy-5a-androstane	Lipid	Androgenic steroids	5.84	0.070	−0.15	0.063	−0.20	0.005
Hydrochlorothiazide	Xenobiotics	Drug	5.68	0.078	0.24	0.055	0.37	0.001
Monoglyceride (22:6(4Z,7Z,10Z,13Z,16Z,19Z)/0:0/0:0)	Lipid	Monoacylglycerol	5.63	0.082	0.00	0.994	0.09	0.075
Sphingomyelin (d18:1/22:0)	Lipid	Sphingomyelins	5.62	0.082	−0.07	0.008	−0.08	0.001
Dehydroepiandrosterone (DHEA) sulfate	Lipid	Sterol/steroid	5.56	0.083	−0.09	0.034	−0.13	0.001
L-Cysteine	Amino acid	Cysteine, methionine, SAM, taurine metabolism	5.56	0.083	−0.02	0.406	0.03	0.055
Etiocholanolone glucuronide	Lipid	Androgenic steroids	5.46	0.089	−0.17	0.026	−0.22	0.001
4-Androsten-3alpha,17alpha-diol monosulfate (3) or androstenediol (3alpha, 17alpha) monsulfate (3)	Lipid	Androgenic steroids	5.46	0.089	−0.10	0.050	−0.14	0.003
Lidocaine	Xenobiotics	Drug	5.42	0.091	0.18	0.001	0.12	0.017
Ergothioneine	Xenobiotics	Food component/plant	5.38	0.091	−0.05	0.224	0.06	0.118
Gamma-Glutamylalanine	Peptide	Gamma-glutamyl	5.37	0.091	0.03	0.300	0.02	0.353
Pioglitazone	Xenobiotics	Drug—metabolic	5.37	0.091	0.60	0.001	0.38	0.018
Phenol sulfate	Amino acid	Phenylalanine and tyrosine metabolism	5.31	0.092	0.02	0.742	−0.09	0.024
5′-Methylthioadenosine	Amino acid	Polyamine metabolism	5.25	0.096	0.14	0.001	0.08	0.033
Ketopioglitazone	Xenobiotics	Drug—metabolic	5.23	0.098	0.20	0.001	0.12	0.020
Hydroxypioglitazone (M-IV)	Xenobiotics	Drug—metabolic	5.22	0.098	0.12	0.001	0.07	0.021
Phosphatidylcholine (18:2(9Z,12Z))	Lipid	Lysolipid	5.18	0.099	−0.07	0.001	−0.03	0.055

Abbreviations: PLCO, Prostate, Lung, Colorectal, and Ovarian Cancer Screening Trial; CEHC, carboxyethylhydroxychroman. ^1^ Estimates and *p*-values were derived using ANCOVA with the 0 IU/day as the reference group. Metabolites were ordered by descending statistical significance of FDR-adjusted *p*-values and alphabetically if they had the same *p*-value. ^2^ Adjusted for age, sex, body weight, smoking status, and multivitamin use.

**Table 3:** Metabolites associated with daily supplemental vitamin E intake according to sex in the PLCO Study (FDR-adjusted *p*-value <0.1) ^1,2^.

	Men (*n* = 1837)					Women (*n* = 1572)				
		4.3–<400 IU		≥400 IU			4.3–<400 IU		≥400 IU	
Metabolite	FDR- Adjusted *p*-Value	Effect Size (β)	*p*-Value	Effect Size (β)	*p*-Value	FDR- Adjusted *p*-Value	Effect Size (β)	*p*-Value	Effect Size (β)	*p*-Value
Alpha-CEHC sulfate	<0.001	0.94	<0.001	10^−18^	<0.001	<0.001	1.26	<0.001	2.69	<0.001
Alpha-CEHC glucuronide	<0.001	0.47	<0.001	1.50	<0.001	<0.001	0.90	<0.001	2.29	<0.001
Alpha-Tocopherol	<0.001	0.16	<0.001	0.43	<0.001	<0.001	0.19	<0.001	0.45	<0.001
Alpha-CEHC	<0.001	0.48	<0.001	1.37	<0.001	<0.001	0.78	<0.001	2.15	<0.001
Pantothenic acid	<0.001	0.25	<0.001	0.34	<0.001	<0.001	0.10	0.034	0.24	<0.001
4-Pyridoxic acid	<0.001	0.18	0.018	0.42	<0.001	<0.001	0.12	0.222	0.45	<0.001
L-Threonic acid	<0.001	0.15	0.007	0.29	<0.001	<0.001	0.07	0.118	0.24	<0.001
Glyceric acid	<0.001	0.10	0.001	0.15	<0.001	<0.001	0.06	0.025	0.11	<0.001
Delta-Tocopherol	<0.001	−0.19	0.176	−0.62	<0.001	<0.001	−0.21	0.019	−0.45	<0.001
Oxalic acid	<0.001	0.11	0.055	0.26	<0.001	<0.001	0.07	0.178	0.21	<0.001
N1-Methyl-4-pyridone-3-carboxamide	0.003	0.11	0.027	0.20	<0.001	0.074	0.04	0.468	0.15	0.002
Pyridoxal	0.010	−0.01	0.916	0.29	0.002	<0.001	0.12	0.472	0.61	<0.001
Glycocholenate sulfate	0.052	−0.08	0.055	−0.13	<0.001	0.009	−0.13	0.004	−0.17	<0.001
C_22_ lactone sulfate (X_12063) ^3^	0.595	0.02	0.814	−0.08	0.188	0.046	−0.12	0.186	−0.26	0.001
5-Androstenediol ^3^	0.305	−0.05	0.253	−0.11	0.011	0.057	−0.15	0.023	−0.21	<0.001
Androsterone glucuronide ^3^	0.531	−0.06	0.343	−0.11	0.048	0.091	−0.32	0.010	−0.37	0.001

Abbreviations: FDR, false discovery rate; CEHC, carboxyethylhydroxychroman. ^1^ Beta-estimates and *p*-values were derived using ANCOVA with the 0 IU intake/day as the reference group. ^2^ Adjusted for age, sex, body weight, smoking status, and multivitamin use. ^3^ Metabolite significant in women (FDR < 0.1) but not in men.

## Data Availability

The data used in this study can be obtained from the PLCO website (https://cdas.cancer.gov/learn/plco/instructions/ (accessed on 16 June 2023)).
